# Needle-Free Injection Enhances the Immunogenicity and Antitumor Efficacy of Whole-Cell Tumor Vaccines

**DOI:** 10.3390/vaccines14050392

**Published:** 2026-04-27

**Authors:** Chin-Yang Chang, Yu-Diao Kuan, Jiayu A. Tai, Nan Ju, Yen-Liang Li, Munehisa Shimamura

**Affiliations:** 1 Department of Gene and Stem Cell Regenerative Therapeutics, Graduate School of Medicine, Osaka University, Osaka 565-0871, Japan; 2 Department of Device Application for Molecular Therapeutics, Graduate School of Medicine, Osaka University, Osaka 565-0871, Japan; 3 National Institute of Cancer Research, National Health Research Institutes, Tainan 704, Taiwan

**Keywords:** needle-free injection, whole-cell tumor vaccine, cancer immunotherapy, cancer vaccine, antitumor immunity

## Abstract

Background/Objectives: Whole-cell vaccines have demonstrated clinical potential in cancer treatment and recurrence prevention, yet their immunogenicity and dendritic cell (DC) activation remain suboptimal. This study aimed to evaluate whether a needle-free injector (NFI) could enhance the immunogenicity and antitumor efficacy of whole-cell tumor vaccines. Methods: Adaptive immune responses induced by NFI and traditional syringe injection (SYI) were compared following whole-cell vaccine administration. The morphology of vaccine fluid ejected by NFI and SYI was examined, and the effects on DC antigen uptake and activation were assessed. Antitumor efficacy was further evaluated in MC38 colon adenocarcinoma challenge models. Results: NFI administration elicited stronger antigen-specific adaptive immune responses than SYI. The high-velocity pressure generated by NFI resulted in fragmentation of whole-cell vaccine material, and this morphological alteration was associated with enhanced DC antigen uptake and activation. These immunological improvements corresponded with superior tumor suppression in MC38 models following NFI-delivered vaccination. Conclusions: NFI delivery enhances the immunogenicity and antitumor efficacy of whole-cell tumor vaccines. These findings suggest that needle-free injectors may serve as a simple and effective strategy to improve the performance of whole-cell cancer vaccines.

## 1. Introduction

Cancer remains one of the leading causes of mortality worldwide, accounting for millions of deaths annually despite significant advancements in medical research and therapeutic strategies [1]. Among the various approaches to cancer therapy, cancer vaccines represent a promising immunotherapeutic option that harnesses the body’s adaptive immune system to generate cancer antigen-specific immune responses [2,3]. Unlike conventional therapies that directly eliminate cancer cells through cytotoxic mechanisms, cancer vaccines stimulate immune cells to recognize and destroy malignant cells, offering a unique and potentially long-lasting therapeutic strategy [2,4].

Within the scope of cancer vaccines, whole-cell vaccines have garnered particular attention due to their ability to present a wide array of tumor-associated antigens (TAAs), enabling a more comprehensive and polyclonal immune response compared with single-antigen approaches [5,6]. Although neoantigens are recognized as critical determinants of cancer-specific immunity, their highly individualized nature poses substantial challenges to the development of universally applicable therapeutic strategies [7]. Furthermore, antigen escape, whereby tumor cells downregulate or lose specific antigenic targets under immune pressure, highlights the vulnerability of single-antigen or neoantigen-focused approaches [4,7]. In this context, the broad antigenic coverage afforded by whole-cell vaccines, encompassing both shared TAAs and patient-specific neoantigens, renders their immunotherapeutic potential particularly compelling [5,7].

Whole-cell vaccines can be classified into two major categories based on their cellular origin: autologous and allogeneic [5,6]. Autologous vaccines, derived directly from a patient’s resected tumor tissue, offer complete immunological compatibility; however, their clinical application is often limited by the requirement for surgical tumor resection, which may not be feasible for all patients [8]. Allogeneic whole-cell vaccines, sourced from established tumor cell lines, provide a more accessible and scalable alternative, particularly when genetically modified to enhance immunogenicity [8,9]. For example, the GVAX platform incorporates the granulocyte–macrophage colony-stimulating factor (GM-CSF) gene into irradiated allogeneic tumor cells, enabling local GM-CSF secretion upon administration [10,11], which subsequently recruits and activates antigen-presenting cells (APCs) at the injection site [10,12]. The clinical promise of whole-cell vaccines extends beyond active treatment to recurrence prevention; notably, OncoVAX demonstrated significant benefit in randomized phase III trials for stage II colon cancer [13]. Despite these encouraging outcomes, the overall therapeutic efficacy of whole-cell vaccines remains suboptimal across broader patient populations, underscoring the need for improved delivery strategies [2,5].

Recent advances in vaccine technology have highlighted innovative delivery systems, with needle-free injectors emerging as a particularly promising platform. These devices utilize high-pressure mechanical propulsion to deliver vaccines intradermally without needles, reducing patient discomfort and minimizing needle-associated complications [14,15]. In recent years, needle-free injectors have been increasingly applied to nucleic acid–based vaccines and gene-delivery platforms, demonstrating favorable safety and robust immunogenicity in infectious disease prevention, including COVID-19 [14,15,16,17]. Beyond infectious diseases, needle-free injectors have also been applied in cell engineering, such as generating tumor–dendritic cell fusion vaccines [18]. Importantly, the high-pressure delivery may augment the release of damage-associated molecular patterns (DAMPs) from cells undergoing immunogenic cell death, while the resulting mechanical fragmentation of cellular debris may enhance phagocytic uptake by APCs, collectively amplifying innate and adaptive immune responses [19,20]. Nevertheless, whole-cell vaccines are still predominantly administered via conventional needle-based injections, which may limit their full immunotherapeutic potential [6,21].

Activation of APCs, particularly dendritic cells (DCs), is critical for whole-cell vaccine-mediated immunity [22,23]. Upon antigen capture and processing, mature DCs migrate to draining lymph nodes, where they prime and expand tumor-specific cytotoxic T lymphocytes (CTLs) capable of targeted cancer cell destruction [22,23,24]. In this study, we investigated whether pressure-based needle-free injector delivery could enhance the efficacy of whole-cell vaccines by improving antigen uptake, DC activation, and downstream CTL responses, with the ultimate goal of expanding the clinical applicability of whole-cell vaccines in oncology.

## 2. Materials and Methods

### 2.1. Animals and Cell Lines

Six-week-old female BALB/c mice and C57BL/6N mice (both from CLEA Japan Inc., Tokyo, Japan) were used throughout this study. All animals were maintained and handled in accordance with the approved protocols and guidelines of the Animal Committee of Osaka University (Suita, Japan). All animal experiments were approved by the Institute of Experimental Animal Sciences, Faculty of Medicine, Osaka University (Approval No. 03-004-020). Animals were euthanized by carbon dioxide inhalation following institutional guidelines.

The 4T1 mammary carcinoma cell line was maintained in Roswell Park Memorial Institute 1640 (RPMI1640) medium (Nacalai Tesque Inc., Kyoto, Japan), and the MC38 colon adenocarcinoma cell line was maintained in Dulbecco’s Modified Eagle Medium (DMEM) (Nacalai Tesque Inc., Kyoto, Japan). Both media were supplemented with 10% fetal bovine serum (BioWest, Nuaillé, France) and 0.1 mg·mL^−1^ penicillin–streptomycin solution (Nacalai Tesque Inc.). All cell lines were cultured at 37 °C in a humidified atmosphere containing 5% CO_2_.

### 2.2. Splenocyte, Macrophage, and Dendritic Cell Preparation

Spleens were harvested from naïve C57BL/6N and BALB/c mice. Single-cell suspensions were prepared by passing the tissue through a 40 μm mesh sieve, followed by red blood cell lysis using hemolysis buffer (Immuno-Biological Laboratories Co., Ltd., Gunma, Japan). Bone marrow-derived macrophages (BMDMs) were generated as follows. Bone marrow cells were seeded at 5 × 10^5^ cells·mL^−1^ in complete RPMI1640 medium (supplemented with 10% FBS and 1% penicillin–streptomycin) containing 10% L929-conditioned medium and cultured for 7 days at 37 °C with 5% CO_2_. At the end of the differentiation period, all adherent and non-adherent cells were harvested for downstream experiments.

Bone marrow-derived dendritic cells (BMDCs) were prepared by flushing the bone marrow from the tibiae and femora with RPMI1640 medium, followed by filtration through a 40 μm mesh sieve. After washing, bone marrow cells were seeded in complete RPMI1640 medium supplemented with 10 ng·mL^−1^ recombinant mouse GM-CSF (BioLegend, San Diego, CA, USA), as described previously [25]. Culture medium was replaced on Days 2 and 4. On Day 6, non-adherent and loosely adherent proliferating cells were harvested and confirmed as DCs by flow cytometric analysis of CD11c expression.

### 2.3. Whole-Cell Vaccine Preparation

Whole-cell vaccines were prepared by either heat or radiation inactivation of tumor cells. For heat inactivation, cells were incubated at 45 °C for 30 min using a dry bath. For radiation inactivation, cells were exposed to 50 Gy using a Gammacell 40 Exactor irradiator (MDS Nordion, Ottawa, ON, Canada). Following inactivation, cells were returned to a 37 °C, 5% CO_2_ incubator for 20 h. Cells were then collected, resuspended in CellBanker freezing medium, and stored at −80 °C until use.

Prior to use, cryopreserved whole-cell vaccines were thawed in a 37 °C water bath for 90 s, then washed twice with phosphate-buffered saline (PBS) using a 10-fold volume excess and centrifuged at 400× *g* for 5 min per wash. To generate ejected whole-cell vaccine fractions, the washed vaccine was loaded into either a conventional syringe or a needle-free injection device (Daicel, Osaka, Japan). The loaded vaccine was then ejected, and the resulting material was collected by centrifugation at 800× *g* for 1 min. The resulting supernatant and pellet fractions were separately collected for subsequent in vitro experiments and analyses.

### 2.4. Ejected Cell Characterization and Phagocytosis Assay

Ejected whole-cell vaccine fractions were prepared as described in Section 2.3. For characterization, 1 × 10^6^ cells per fraction were analyzed as follows. Physical morphology was assessed by measuring forward scatter (FSC) and side scatter (SSC) parameters using a CytoFLEX XS flow cytometer (Beckman Coulter, Brea, CA, USA). Morphological images were acquired using a BZ-X710 all-in-one fluorescence microscope (Keyence Corporation, Osaka, Japan). Cell viability was determined by 7-aminoactinomycin D (7-AAD) exclusion staining, analyzed by flow cytometry.

For the phagocytosis assay, whole-cell vaccines were labeled with the lipophilic fluorescent dye DiR (1,1′-dioctadecyl-3,3,3′,3′-tetramethylindotricarbocyanine iodide) prior to ejection. DiR-labeled vaccines were processed by either needle-free injection device (PJI) or conventional syringe (SYR) as described above, and the resulting ejected fractions were co-cultured with BMDCs or BMDMs for 24 h. Following co-culture, phagocytic uptake was quantified by flow cytometry using a CytoFLEX XS. DCs were identified as CD11c^+^ cells using anti-CD11c antibody (clone N418, BioLegend), and macrophages were identified as CD11b^+^F4/80^+^ cells using anti-CD11b (clone M1/70, BioLegend) and anti-F4/80 (clone BM8, BioLegend) antibodies. Phagocytosis was defined as the percentage of CD11c^+^DiR^+^ cells among the DC population and CD11b^+^F4/80^+^DiR^+^ cells among the macrophage population.

### 2.5. Splenocyte Activation and ELISpot Assay

Spleens were harvested from DC–tumor cell fusion vaccine-treated mice 12 days after the final vaccination. Splenocytes were isolated as described in Section 2.2. This time point is widely used to capture peak vaccine-induced adaptive immune responses in murine models [26]. To prepare stimulator cells, MC38 colon adenocarcinoma cells were treated with 15 μg·mL^−1^ mitomycin C (Nacalai Tesque Inc.) for 45 min to inhibit proliferation. Splenocytes and mitomycin C-treated MC38 cells were mixed at a 10:1 ratio and co-cultured for 48 h. Non-adherent splenocytes were subsequently collected, and IFN-γ secretion was assessed using the Mouse IFN-γ ELISpot Development Module and the ELISpot Blue Color Module (both from R&D Systems, Minneapolis, MN, USA) according to the manufacturer’s instructions [18]. IFN-γ-secreting spot-forming cells were enumerated and recorded. ELISpot assays were performed in triplicate wells for each mouse, and spot numbers were averaged across wells after background subtraction.

### 2.6. Tumor Challenge Model

Mice were inoculated subcutaneously with 2 × 10^5^ tumor cells on Day 0. The first vaccination was administered intradermally at a dose of 1 × 10^6^ cells on Day 1, and the second vaccination was administered by the same route and dose on Day 5. Tumor dimensions were measured every 2–3 days using digital calipers. Tumor volume was calculated using the formula:

V = W2 × L2V = 2W2 × L

where L is the longest diameter, and W is the shortest diameter of the tumor.

### 2.7. Inflammation Analysis by Quantitative Real-Time PCR

Tissue samples for innate immune profiling were collected 24 h after vaccine administration to assess early local immune responses at the injection site. Total RNA was extracted from the injection-site tissue using the Maxwell RSC simplyRNA Tissue Kit (Promega, Madison, WI, USA) according to the manufacturer’s instructions. Complementary DNA synthesis was performed using the ReverTra Ace reverse transcriptase kit (TOYOBO Co., Ltd., Osaka, Japan). Quantitative real-time PCR (qRT-PCR) was carried out using SYBR Green PCR reagent (TOYOBO Co., Ltd.). All primer sequences used in this study are listed in Table S1. β-actin was used as the housekeeping reference gene, and relative gene expression levels were calculated using the 2^(−ΔΔCT)^ method.

### 2.8. Western Blot

Ejected whole-cell vaccine fractions were lysed in RIPA lysis buffer (Nacalai Tesque Inc.) to extract total protein. Proteins were separated by 10% SDS-PAGE and transferred onto polyvinylidene fluoride (PVDF) membranes (Sigma-Aldrich, St. Louis, MO, USA) (Cat. No. 3010040001). Membranes were blocked with blocking buffer (Nacalai Tesque Inc.) for 30 min at room temperature, then incubated with primary antibodies for 1 h at room temperature. The following primary antibodies were used: anti-HSP70–HRP (clone W27, BioLegend) and anti-β-actin (clone 8H10D10, Cell Signaling Technology, Danvers, MA, USA). Membranes were subsequently incubated with HRP-conjugated secondary antibodies for 1 h at room temperature, followed by five washes with PBST. Bands were visualized using the Western Bright ECL detection system (Nacalai Tesque Inc.), and band intensities were quantified using ImageJ software 1.53a (NIH, Bethesda, MD, USA).

### 2.9. Statistical Analysis

All statistical analyses were performed using GraphPad Prism 9 (GraphPad Software, Boston, MA, USA). Group comparisons were conducted using a two-tailed unpaired Student’s *t*-test. A *p*-value < 0.05 was considered statistically significant.

## 3. Results

### 3.1. Injection Method Influences the Immunogenicity of Whole-Cell Vaccines

**Figure 1:**
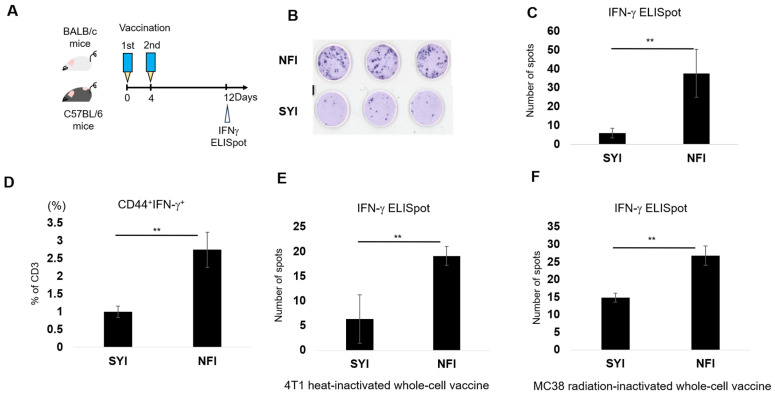
Needle-free injection enhances the immunogenicity of whole-cell cancer vaccines across tumor models and inactivation methods. (**A**) Schematic of the vaccination and analysis workflow. Naïve mice received whole-cell vaccines delivered by needle-free injection (NFI) or syringe injection (SYI) on Day 0 and Day 4. Immune responses were assessed on Day 12 by IFN-γ ELISpot or by T-cell restimulation followed by flow cytometry. (**B**) IFN-γ ELISpot assay setup. Each well was seeded with splenocytes and stimulated with heat-inactivated tumor cells. Colorimetric development was performed after 48 h. (**C**) MC38 heat-inactivated whole-cell vaccine (45 °C for 30 min; cells per dose) was administered twice to C57BL/6 mice. Antigen-specific IFN-γ responses were quantified by ELISpot on Day 12 (*n* = 3). (**D**) Flow cytometric analysis of tumor-specific T-cell activation. Splenocytes collected on Day 12 from MC38-vaccinated C57BL/6 mice were restimulated with 10% inactivated MC38 cells and analyzed for the frequency of CD3^+^CD44^+^IFN-γ^+^ T cells (*n* = 4). (**E**) 4T1 heat-inactivated whole-cell vaccine (45 °C for 30 min) was administered to BALB/c mice, followed by ELISpot quantification of adaptive immune responses on Day 12 (*n* = 3). (**F**) MC38 radiation-inactivated whole-cell vaccine (50 Gy; cells per dose) was administered twice according to the vaccination schedule, and antigen-specific responses were assessed by ELISpot (*n* = 3). All data are presented as mean ± SEM. Statistical significance was determined using unpaired Student’s *t*-test.; ** *p* < 0.01.

To evaluate whether needle-free injection enhances the immunogenicity of whole-cell vaccines, we compared immune responses induced by NFI and SYI using heat-inactivated MC38 whole-cell vaccines. Mice were vaccinated twice, and antigen-specific responses were assessed by ELISpot assays (Figure 1A–C). NFI elicited a markedly stronger response, with a 6.2-fold increase in IFN-γ spot-forming cells compared with SYI (37.6 vs. 6.0 spots; Figure 1C). Flow cytometric analysis further confirmed enhanced T-cell activation, as the NFI group exhibited significantly higher frequencies of CD44^+^IFN-γ^+^ tumor-specific T cells than the SYI group (Figure 1D).

To characterize the early local immune microenvironment at the injection site, total RNA was extracted from tissue samples collected 24 h after vaccination, and immune-related gene expression was quantified by RT-qPCR. CD11c expression remained low in both groups, indicating minimal dendritic cell accumulation regardless of injection method. Although CD45 expression showed no significant difference, CD11b mRNA levels were 3.6-fold higher in the NFI group (Supplementary Figure S1), suggesting increased recruitment of monocyte-lineage myeloid cells to the injection site.

We next examined whether the enhanced immunogenicity conferred by NFI was generalizable across tumor models. Using a 4T1 breast cancer whole-cell vaccine, NFI again induced significantly stronger antigen-specific responses than SYI (Figure 1E), indicating that this effect is not restricted to a specific cancer type. To determine whether NFI efficacy depends on the method of tumor-cell inactivation, we tested radiation-inactivated MC38 vaccines. ELISpot assays showed that NFI increased IFN-γ responses by 1.8-fold relative to SYI (Figure 1F), demonstrating that the immunostimulatory advantage of NFI extends beyond heat-inactivated vaccines.

### 3.2. Impact of Injectors on Whole-Cell Vaccines

Given that modifying the delivery method markedly enhanced vaccine efficacy, we next investigated how the physical mechanism of NFI affects whole-cell vaccine preparations. Needle-free injectors generate a high-velocity jet that delivers formulations intradermally without a needle [27]. Because whole-cell vaccines contain intact cells—substantially larger than nucleic acids or protein subunits—the high-pressure jet may impose mechanical stress sufficient to disrupt cellular integrity.

**Figure 2:**
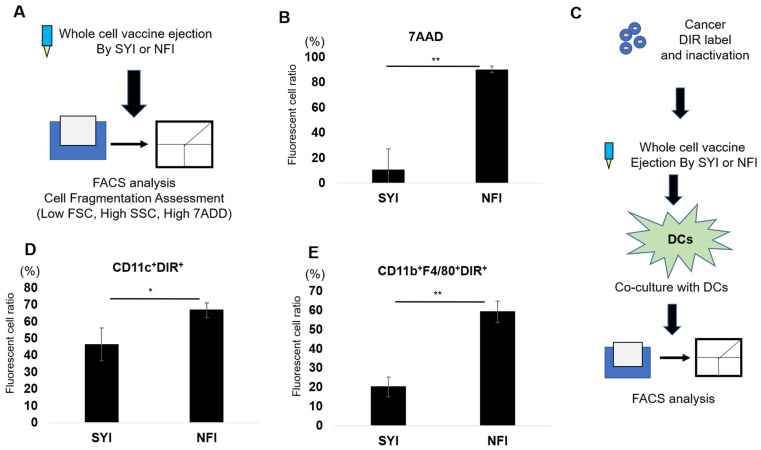
Needle-free injection disrupts whole-cell vaccine integrity and enhances uptake by antigen-presenting cells. (**A**) Whole-cell vaccines were loaded into either the needle-free injector (NFI) or syringe injector (SYI) and ejected into collection tubes. The resulting cell suspensions were analyzed by flow cytometry to assess cell size and fragmentation using forward scatter (FSC) and side scatter (SSC) parameters. Membrane damage was evaluated using 7-AAD staining. (**B**) Membrane integrity of NFI- and SYI-ejected cells was quantified by 7-AAD staining followed by flow cytometric analysis. (*n* = 3) (**C**) Uptake of NFI- or SYI-ejected cells by antigen-presenting cells. Ejected cells were labeled with DIR and co-cultured with dendritic cells (DCs) or macrophages for 24 h. APCs that acquired ejected cells were identified as double-positive populations (DCs: CD11c^+^DIR^+^; macrophages: CD11b^+^F4/80^+^DIR^+^) located in the Q1-UR quadrant. (**D**) Quantification of DC uptake of NFI- or SYI-ejected cells (CD11c^+^DIR^+^; *n* = 5). (**E**) Quantification of macrophage uptake of NFI- or SYI-ejected cells (CD11b^+^F4/80^+^DIR^+^; *n* = 4). All data are presented as mean ± SEM. Statistical significance was determined using unpaired Student’s *t*-test. * *p* < 0.05; ** *p* < 0.01.

To test this, we collected vaccine fluid ejected by NFI or SYI and analyzed cell size and granularity by flow cytometry (FSC/SSC) (Figure 2A and Supplementary Figure S3A). NFI-processed cells (NFI-C) exhibited markedly reduced FSC and SSC signals compared with SYI-processed cells (SYI-C), indicating extensive fragmentation into smaller particles (Supplementary Figure S2). Membrane integrity analysis using 7-AAD staining further supported this observation: 90.2% of NFI-C were 7-AAD–positive compared with 10.9% of SYI-C (Figure 2B; *p* < 0.05), demonstrating that NFI induces substantial membrane disruption and generates non-intact, irregular cell fragments rather than morphologically preserved cells (Supplementary Figure S4).

### 3.3. Phagocytic Uptake of NFI- and SYI-Processed Vaccine Material

NFI processing alters both the morphology and size of whole-cell vaccines, generating smaller and more fragmented particles than SYI-processed cells (Supplementary Figure S2). Because particle size and shape strongly influence phagocytosis by antigen-presenting cells (APCs) [28], we hypothesized that these NFI-induced physical changes could modulate how APCs internalize vaccine material. We therefore examined whether NFI-mediated disruption affects antigen uptake.

Dendritic cells (DCs), as professional APCs, play a central role in engulfing exogenous antigens and initiating adaptive immunity. To assess whether NFI-processed material alters phagocytic efficiency, tumor cells were labeled with DiR prior to vaccine preparation, delivered by either NFI or SYI, and subsequently co-cultured with DCs for uptake analysis (Figure 2C and Supplementary Figure S3B). After 24 h, flow cytometric quantification of CD11c^+^DiR^+^ cells revealed that DCs exposed to NFI-processed cells (NFI-C) exhibited significantly higher antigen uptake than those exposed to SYI-processed cells (SYI-C) (Figure 2D). A similar enhancement was observed in macrophages, another major APC population, where NFI-C induced greater antigen uptake than SYI-C (Figure 2E). Together, these findings demonstrate that NFI enhances antigen phagocytosis by APCs, likely due to the increased fragmentation and altered physical properties of NFI-processed whole-cell vaccines.

### 3.4. Immunostimulatory Effects of NFI-Processed Cell Lysates on APCs

Whole-cell vaccines rely on antigen-presenting cells (APCs), such as dendritic cells (DCs), to internalize tumor-derived material and initiate adaptive immune responses. In addition to antigen uptake, APC activation can be triggered by danger-associated molecular patterns (DAMPs) released from stressed or damaged cells [29]. Our earlier findings showed that NFI mechanically disrupts whole-cell vaccines, generating smaller, fragmented particles and enhancing their phagocytosis by APCs. However, increased antigen uptake does not necessarily translate into APC activation [30], and whether NFI-processed material (NFI-C) also promotes APC activation remained unclear.

**Figure 3:**
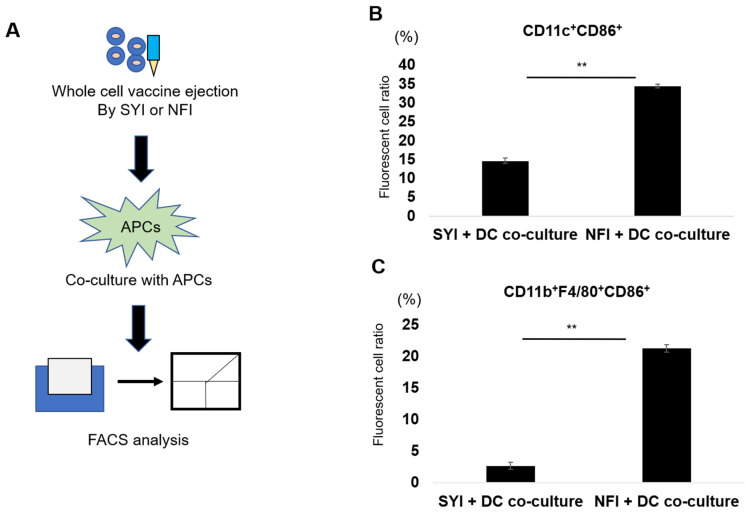
NFI-processed cell lysates enhance activation of dendritic cells and macrophages. (**A**) Experimental workflow for assessing APC activation. Tumor cells were inactivated as whole-cell vaccines, labeled with DIR, and ejected using either syringe injection (SYI) or needle-free injection (NFI). The resulting cell lysates were co-cultured with dendritic cells (DCs) or macrophages for 24 h, followed by flow cytometric analysis of activation marker expression. (**B**) DC activation following exposure to NFI- or SYI-processed cell lysates. Ejected cell suspensions (1 × 10^6^ cells) were co-cultured with 5 × 10^5^ DCs for 24 h, and the proportion of activated DCs (CD11c^+^CD86^+^) was quantified by flow cytometry (*n* = 3). (**C**) Macrophage activation following exposure to NFI- or SYI-processed cell lysates (*n* = 4). Ejected cell suspensions (1 × 10^6^ cells) were co-cultured with 5 × 10^5^ macrophages for 24 h, and activated macrophages (CD11b^+^F4/80^+^CD86^+^) were quantified by flow cytometry. All data are presented as mean ± SEM. Statistical significance was determined using unpaired Student’s *t*-test. ** *p* < 0.01.

To determine whether NFI-C stimulates DC activation, we co-cultured DCs with cell lysates obtained from NFI or SYI and assessed expression of the activation marker CD86 on CD11c^+^ cells after 24 h (Figure 3A and Supplementary Figure S3C). DCs exposed to NFI-C exhibited markedly higher activation than those exposed to SYI-C, showing approximately a four-fold increase in CD11c^+^CD86^+^ cells (Figure 3B). A similar enhancement was observed in macrophages, where NFI-C induced greater antigen uptake and higher expression of activation markers compared with SYI-C (Figure 3C). These results suggest that, beyond enhancing antigen phagocytosis, NFI processing may generate more immunostimulatory cell-derived material by mechanically disrupting pre-inactivated tumor cells, thereby facilitating the release and exposure of pre-existing DAMP-containing fragments to APCs.

### 3.5. NFI Induces Protein Release and Enhances APC Activation

Previous studies have shown that APC activation following phagocytosis depends not only on the amount of internalized material but also on the intrinsic properties and stress status of the engulfed cells [31]. During whole-cell vaccine preparation, tumor cells undergo inactivation procedures—such as heat or irradiation—that induce cellular stress and promote the accumulation of danger-associated molecular patterns (DAMPs), which can further stimulate immune activation [32].

**Figure 4:**
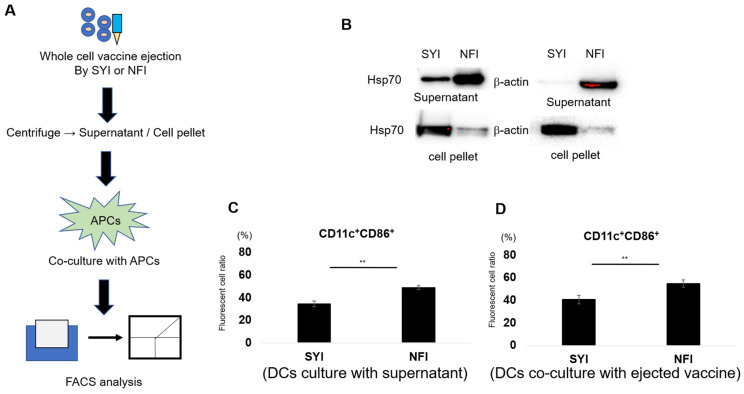
Needle-free injection promotes intracellular protein release and enhances dendritic cell activation. (**A**) Experimental workflow for separating supernatant and cell pellet from NFI- or SYI-processed whole-cell vaccines. Tumor cells were inactivated, loaded into either the syringe injector (SYI) or needle-free injector (NFI), and ejected into collection tubes. The resulting suspensions were centrifuged to obtain supernatant and cell pellet fractions, which were subsequently used for APC stimulation assays. (**B**) Western blot analysis of intracellular protein release following NFI or SYI processing. Hsp70 and β-actin levels were examined in the supernatant (top panels) and cell pellet (bottom panels). NFI-processed samples showed increased protein release into the supernatant compared with SYI-processed samples. (**C**) Dendritic cell activation induced by supernatants from NFI- or SYI-processed vaccines. Supernatants were co-cultured with DCs for 24 h, and activated DCs (CD11c^+^CD86^+^) were quantified by flow cytometry (*n* = 4). (**D**) Dendritic cell activation induced by cell pellets from NFI- or SYI-processed vaccines. Cell pellets were co-cultured with DCs for 24 h, and activation was assessed by the proportion of CD11c^+^CD86^+^ cells (*n* = 4). All data are presented as mean ± SEM. Statistical significance was determined using unpaired Student’s *t*-test.; ** *p* < 0.01.

Our earlier findings demonstrated that NFI causes extensive cell fragmentation and membrane disruption, allowing 7-AAD penetration and suggesting that NFI may facilitate the release of intracellular proteins and DAMP-associated molecules already present within pre-inactivated tumor cells. To test this possibility, we separated the supernatant and cell pellet from NFI- and SYI-processed cell suspensions and analyzed protein distribution by Western blotting (Figure 4A).

Western blot analysis revealed that the NFI-C supernatant contained markedly higher levels of Hsp70 compared with the SYI-C supernatant, whereas the corresponding NFI-C pellet retained only minimal Hsp70 (Figure 4B). In contrast, SYI-processed samples showed the opposite pattern, with most Hsp70 remaining in the pellet and only a small fraction detected in the supernatant. A similar distribution was observed for β-actin, indicating that NFI processing causes intracellular proteins to leak into the extracellular fraction due to membrane disruption.

We next examined whether this protein-rich supernatant contributes to APC activation. DCs were co-cultured with either the supernatant or pellet from NFI- or SYI-processed samples (Figure 4A), and activation was assessed by CD86 expression on CD11c^+^ cells. Both the NFI-C supernatant and NFI-C pellet induced significantly higher DC activation compared with their SYI counterparts (Figure 4C,D).

Together, these results suggest that NFI not only increases antigen fragmentation and phagocytosis but also enhances the release and exposure of intracellular proteins and DAMP-associated components from pre-inactivated tumor cells, generating a more immunostimulatory vaccine preparation than conventional syringe injection.

### 3.6. Therapeutic Effect in Tumor Challenge Models

The above findings demonstrate that NFI processing enhances antigen uptake and activation of antigen-presenting cells (APCs), suggesting a potential improvement in adaptive immune responses. However, whether these immunological enhancements translate into superior therapeutic efficacy remained unclear. To address this, we evaluated the anti-tumor activity of NFI-processed whole-cell vaccines in a tumor challenge model.

**Figure 5:**
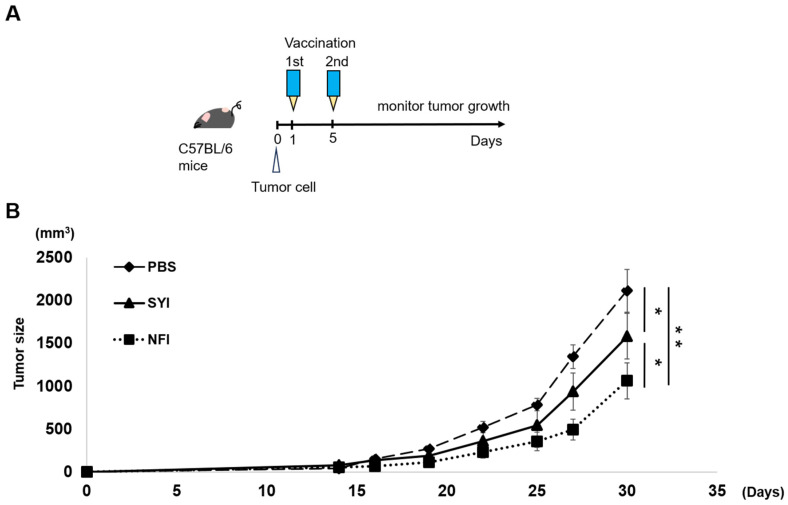
Needle-free injection enhances the therapeutic efficacy of whole-cell cancer vaccines in a tumor challenge model. (**A**) Experimental design of the tumor challenge model. C57BL/6 mice were inoculated intradermally with 1 × 10^5^ MC38 tumor cells on Day 0. Whole-cell vaccines were administered using either syringe injection (SYI) or needle-free injection (NFI) on Day 1 and Day 5. Tumor growth was monitored thereafter. (**B**) Therapeutic efficacy of SYI- and NFI-processed MC38 whole-cell vaccines. Mice received two doses of whole-cell vaccine (1 × 10^6^ cells per dose), and tumor volume was measured on Day 30 post-tumor inoculation (*n* = 4). NFI-processed vaccines exhibited a greater reduction in tumor growth compared with SYI-processed vaccines. All data are presented as mean ± SEM. Statistical significance was determined using unpaired Student’s *t*-test. * *p* < 0.05; ** *p* < 0.01.

Because whole-cell vaccines function as therapeutic rather than prophylactic vaccines, naïve mice were first inoculated with tumor cells and subsequently administered two vaccine doses, after which tumor growth was monitored (Figure 5A). Both SYI- and NFI-processed vaccines suppressed tumor progression; however, the NFI group exhibited a more pronounced anti-tumor effect, with tumor size reduced by approximately 20% compared with the SYI group (Figure 5B).

These results indicate that the enhanced APC activation and immunostimulatory properties conferred by NFI processing translate into improved therapeutic efficacy, demonstrating that NFI can potentiate the anti-cancer activity of whole-cell vaccines.

## 4. Discussion

Whole-cell vaccines have long been recognized for their broad antigenic coverage and theoretical potential to elicit robust anti-tumor immunity. However, despite decades of development, their clinical performance has remained modest, with limited improvements in patient response rates and overall survival. Several factors contribute to this suboptimal efficacy [33,34,35]. First, many TAAs exhibit inherently low immunogenicity because they originate from self-derived proteins or patient-specific mutations. Second, whole-cell vaccines often lack sufficient adjuvant stimulation to surpass the threshold required for effective DC activation. Third, inadequate antigen uptake and insufficient DC maturation can further limit downstream T-cell priming. These challenges highlight the need for innovative strategies to enhance the immunogenicity of whole-cell vaccines.

Despite these limitations, recent advances have demonstrated that whole-cell vaccines can achieve meaningful clinical benefit when appropriately engineered. For example, the allogeneic GM-CSF–secreting GVAX platform has shown encouraging results in multiple myeloma, achieving complete remission rates exceeding 50%, and has improved overall and disease-free survival in pancreatic ductal adenocarcinoma. Similarly, the autologous whole-cell vaccine OncoVAX demonstrated a 40% reduction in recurrence in stage II colon cancer [10,11,13]. These clinical successes underscore the therapeutic potential of whole-cell vaccines when their immunogenicity is sufficiently enhanced. However, genetic modification strategies such as GM-CSF transduction increase manufacturing complexity and cost, limiting widespread clinical adoption. Therefore, identifying simple, scalable, and cost-effective methods to improve whole-cell vaccine efficacy remains an important unmet need [36,37,38,39].

One promising direction involves leveraging immunogenic cell death (ICD) and the release of DAMPs, such as HMGB1 and heat-shock proteins, which enhance antigenicity and promote DC activation. Heat or radiation inactivation—commonly used in whole-cell vaccine production—induces cellular stress and DAMP accumulation. However, the extent to which these DAMPs are exposed to antigen-presenting cells depends on how the vaccine is delivered [40,41,42]. In this context, NFI, which delivers formulations using high-pressure mechanical force, represents a potentially transformative approach. Although NFI has been widely applied to DNA, RNA, and protein vaccines, its impact on whole-cell vaccines has not been previously explored [27,43].

In this study, we demonstrate that NFI markedly enhances the immunogenicity of whole-cell vaccines compared with conventional SYI. NFI significantly increased antigen-specific T-cell responses across multiple tumor models, including heat-inactivated MC38 and 4T1 vaccines, and also improved responses to radiation-inactivated MC38 vaccines. These findings indicate that the immunological benefits of NFI are not restricted to a specific tumor type or inactivation method and are consistent with previous reports showing that NFI can elicit stronger immune responses than conventional syringe-based delivery [44]. Interestingly, the magnitude of enhancement was lower for radiation-inactivated vaccines, suggesting that the inactivation method may influence the degree of NFI-mediated improvement. One possible explanation is that radiation already induces substantial DAMP release, partially overlapping with the mechanical effects of NFI [45,46].

In our study, we also observed that NFI enhanced adaptive immune activation (Figure 1), and this effect may be linked to early events occurring at the injection site. Although CD11c expression at the injection site remained low 24 h after vaccination in both NFI and SYI groups (Supplementary Figure S1), this likely reflects the rapid migration of activated DCs to draining lymph nodes [47], as reported in previous studies. In contrast, qPCR analysis revealed a markedly higher accumulation of CD11b^+^ myeloid-lineage cells in the NFI group. This suggests that NFI may recruit a larger pool of myeloid precursors to the injection site [48,49], which could subsequently differentiate into additional APC populations, such as DCs or macrophages, thereby supporting sustained antigen presentation and downstream adaptive immunity [50,51]. Although these findings imply that NFI may create a more favorable myeloid environment for initiating immune responses, the precise mechanisms underlying this enhanced recruitment and differentiation require further investigation.

Mechanistically, our data reveal that NFI induces extensive mechanical disruption of whole-cell vaccine preparations. Flow cytometry and microscopy demonstrated that NFI generates smaller, irregular cell fragments with compromised membrane integrity (Supplementary Figures S2 and S3). These physical changes have important immunological consequences. First, smaller antigenic particles are more efficiently internalized by DCs and macrophages, consistent with established principles of size-dependent phagocytosis (Figure 2D,E). Second, Western blot analysis showed that NFI promotes passive leakage of intracellular proteins, including Hsp70, into the extracellular fraction (Figure 4B). Because these proteins are known DAMP-associated molecules generated during the inactivation process, their increased exposure may contribute to enhanced DC activation (Figure 3B). Importantly, our findings do not suggest that NFI induces DAMP production; rather, NFI facilitates the release and presentation of pre-existing DAMPs generated during heat or radiation inactivation.

Consistent with these mechanistic observations, NFI-processed vaccines induced stronger DC activation than SYI-processed vaccines, both in terms of antigen uptake and expression of activation markers. Notably, both the supernatant and pellet fractions of NFI-processed material enhanced DC activation, suggesting that both soluble DAMP-associated components and particulate antigen fragments contribute to the immunostimulatory effect (Figure 4C,D). These results support a model in which NFI enhances whole-cell vaccine efficacy through two complementary mechanisms: (1) generating antigen fragments with more favorable physical properties for phagocytosis, and (2) increasing the extracellular availability of DAMP-associated molecules that promote APC activation.

Importantly, the enhanced innate and adaptive immune responses observed in vitro translated into improved therapeutic efficacy in vivo. In a tumor challenge model designed to mimic clinical conditions, NFI-processed vaccines achieved superior tumor suppression compared with SYI-processed vaccines. This demonstrates that the immunological advantages conferred by NFI are functionally meaningful and can potentiate the therapeutic performance of whole-cell vaccines (Figure 5B). Although our findings highlight the potential of NFI as a simple and scalable strategy to enhance whole-cell vaccine efficacy [43], several considerations remain for clinical translation. Different inactivation methods may interact differently with NFI-mediated mechanical disruption, and the optimal combination of NFI with adjuvants or immunomodulators remains to be determined [27]. Additionally, our study used inactivated cells without supplemental adjuvants; identifying adjuvant strategies that synergize with NFI may further improve vaccine performance. Finally, the extent to which NFI-induced tissue microdamage contributes to local inflammation warrants further investigation [44].

## 5. Conclusions

In conclusion, our study identifies needle-free injection as a powerful yet practical approach to enhance whole-cell vaccine immunogenicity. By mechanically fragmenting tumor cells and increasing the exposure of antigenic and DAMP-associated components, NFI improves APC activation, antigen uptake, and anti-tumor immunity. These findings provide a new perspective on how physical delivery methods shape vaccine immunogenicity and offer a clinically feasible strategy to improve whole-cell cancer vaccines without requiring genetic modification or complex manufacturing processes.

## Data Availability

The data supporting the findings of this study are available from the corresponding author upon reasonable request.

## Supplementary Materials

The following supporting information can be downloaded at: https://www.mdpi.com/article/10.3390/vaccines14050392/s1, Figure S1: Immune-related gene expression after injection; Figure S2: Flow cytometry analysis of cell size and granularity; Figure S3: Flow cytometry gating strategies. (A) Gating strategy for Figure 2A. Cells subjected to mechanical stress were analyzed to assess membrane integrity. The gating strategy identifies 7-AAD–positive cells, representing the population with compromised membrane permeability. (B) Gating strategy for Figure 2C. Phagocytosis was evaluated using dual labeling of cell-specific markers and fluorescently labeled cancer cells. The gating strategy identifies double-positive events corresponding to phagocytic cells. (C) Gating strategy for Figure 3A and Figure 4A. Dendritic cells (DCs) were first identified based on cell surface markers, and this DC population was then analyzed for CD86 expression. The proportion of CD86^+^ DCs is quantified as shown in the main figures; Figure S4: NFI-induced membrane disruption; Table S1: The qRT-PCR primer sequences.

## Abbreviations

The following abbreviations are used in this manuscript:

4T1	4T1 murine mammary carcinoma cell line
7-AAD	7-aminoactinomycin D
APC	antigen-presenting cell
β-actin	beta-actin
CD11b	cluster of differentiation 11b
CD11c	cluster of differentiation 11c
CD44	cluster of differentiation 44
CD45	cluster of differentiation 45
CD86	cluster of differentiation 86
DAMP	danger-associated molecular pattern
DC	dendritic cell
DiR	1,1′-dioctadecyl-3,3,3′,3′-tetramethylindotricarbocyanine iodide
ELISpot	enzyme-linked immunospot assay
FSC	forward scatter
GM-CSF	granulocyte–macrophage colony-stimulating factor
GVAX	GM-CSF–secreting allogeneic whole-cell vaccine platform
HMGB1	high-mobility group box 1
Hsp70	heat-shock protein 70
ICD	immunogenic cell death
IFN-γ	interferon-gamma
MC38	MC38 murine colon adenocarcinoma cell line
NFI	needle-free injector
NFI-C	needle-free injector–processed cells
qPCR	quantitative polymerase chain reaction
RT-qPCR	reverse transcription quantitative polymerase chain reaction
SSC	side scatter
SYI	syringe injector
SYI-C	syringe injector–processed cells
TAA	tumor-associated antigen
