# Supplementary material for: Needle-Free Injection Enhances the Immunogenicity and Antitumor Efficacy of Whole-Cell Tumor Vaccines
Source: Vaccines (Basel). 2026 Apr 27;14(5):392. doi: 10.3390/vaccines14050392 (PMC13211413; doi:10.3390/vaccines14050392)
Supplement: Supplementary file 1 [file vaccines-14-00392-s001.zip › vaccines-4239669-supplementary.pdf]

Table S1. The qRT-PCR primer sequences

	Forward: 5′	Reverse: 5′
beta-actin	GGAGGGGGTTGAGGTGTT	GTGTGCACTTTTATTGGTCTCAAG
CD11c	CCTGAGGGTGGGCTGGAT	GCCAATTTCCTCCGGACAT
CD45	TCAGAAAATGCAACAGTGACAA	CCAACTGACATCTTTCAGGTATG
CD11b	AAACCACAGTCCCGCAGAGA	CGTGTTCACCAGCTGGCTTA

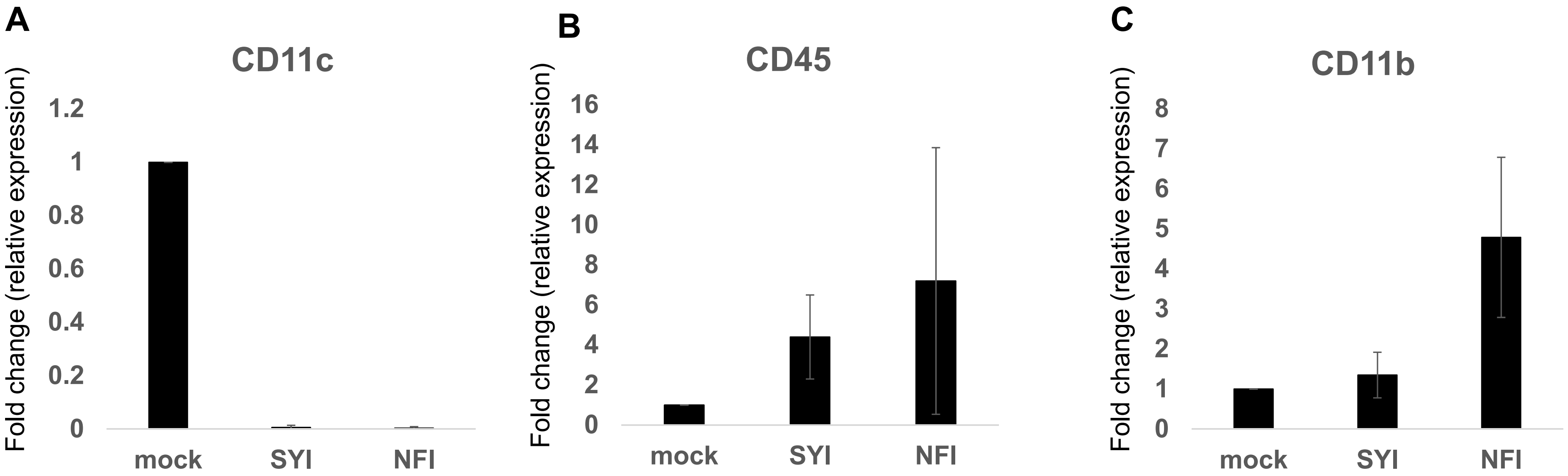

Supplementary Figure S1. Immune-related gene expression after injection.

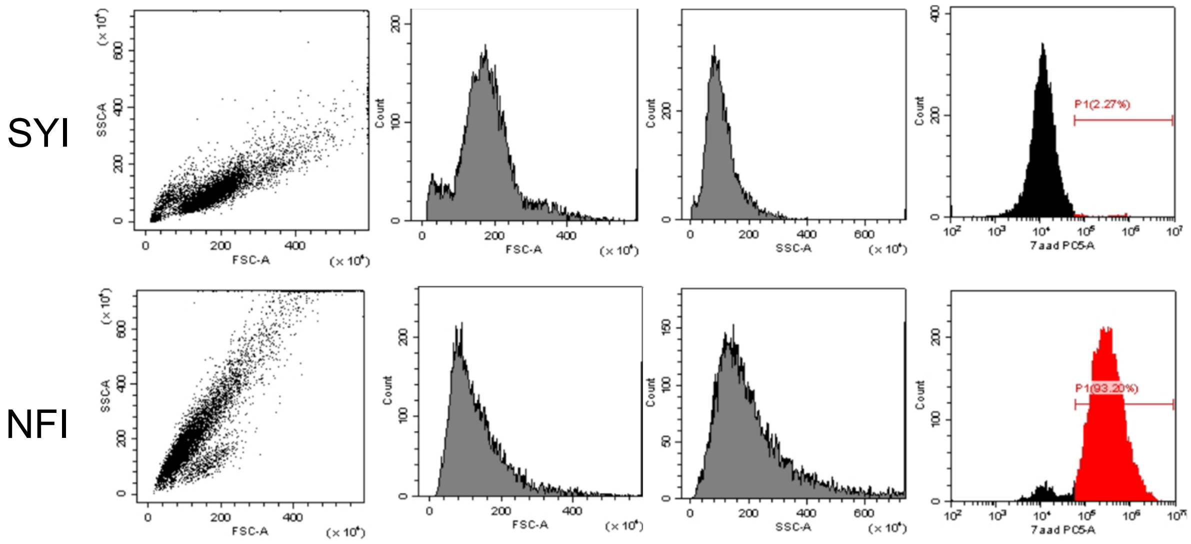

Supplementary Figure S2. Flow cytometry analysis of cell size and granularity.

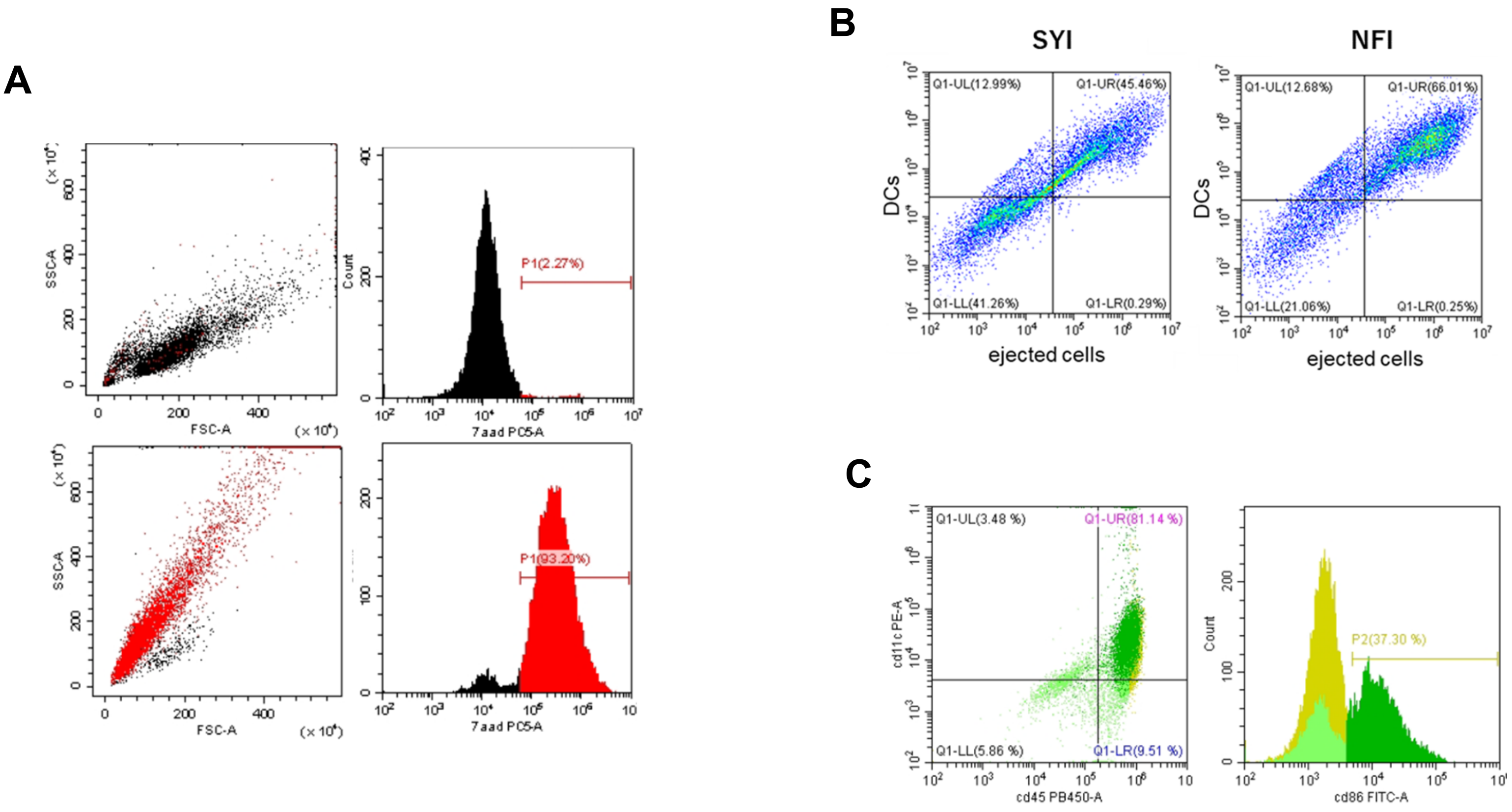

Supplementary Figure S3. Flow cytometry gating strategies.

A. Gating strategy for Figure 2A. Cells subjected to mechanical stress were analyzed to assess membrane integrity. The gating strategy identifies 7-AAD–positive cells, representing the population with compromised membrane permeability.
B. Gating strategy for Figure 2C. Phagocytosis was evaluated using dual labeling of cell-specific markers and fluorescently labeled cancer cells. The gating strategy identifies double-positive events corresponding to phagocytic cells.
C. Gating strategy for Figures 3A and 4A. Dendritic cells (DCs) were first identified based on cell surface markers, and this DC population was then analyzed for CD86 expression. The proportion of $CD86^+$ DCs is quantified as shown in the main figures.

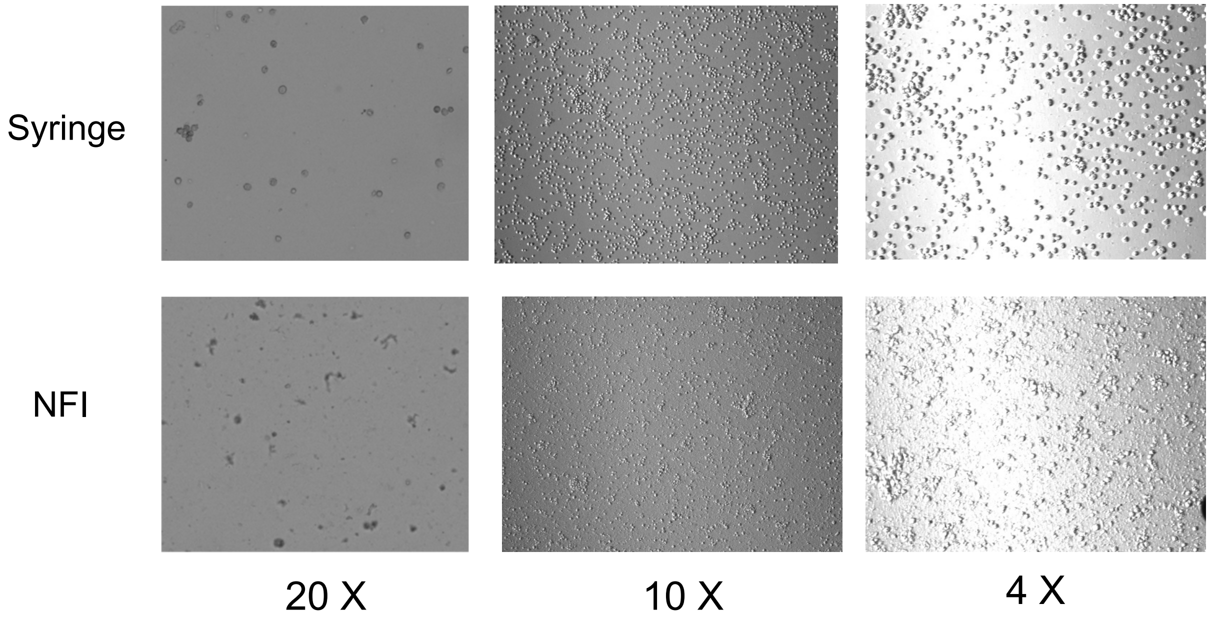

Supplementary Figure S4. NFI-induced membrane disruption.
